# Anle138b modulates α‐synuclein oligomerization and prevents motor decline and neurodegeneration in a mouse model of multiple system atrophy

**DOI:** 10.1002/mds.27562

**Published:** 2018-11-19

**Authors:** Antonio Heras‐Garvin, Daniel Weckbecker, Sergey Ryazanov, Andrei Leonov, Christian Griesinger, Armin Giese, Gregor K. Wenning, Nadia Stefanova

**Affiliations:** ^1^ Department of Neurology, Division of Neurobiology Medical University of Innsbruck Innsbruck Austria; ^2^ NMR Based Structural Biology Max Planck Institute for Biophysical Chemistry Göttingen Germany; ^3^ MODAG GmbH Wendelsheim Germany; ^4^ Center for Neuropathology and Prion Research Ludwig‐Maximilians‐Universität Munich Germany

**Keywords:** α‐synuclein, anle138b, movement disorders, multiple system atrophy, neurodegeneration

## Abstract

**Background:**

MSA is a fatal neurodegenerative disease characterized by autonomic failure and severe motor impairment. Its main pathological hallmark is the accumulation of α‐synuclein in oligodendrocytes, leading to glial and neuronal dysfunction and neurodegeneration. These features are recapitulated in the PLP‐hαSyn mouse model expressing human α‐synuclein in oligodendrocytes. At present, there is no effective disease‐modifying therapy. Previous experiments have shown that the aggregation inhibitor, anle138b, reduces neurodegeneration and behavioral deficits in mouse models of other proteinopathies.

**Objectives:**

To test the therapeutic potential of anle138b in a mouse model of MSA.

**Methods:**

Two‐month‐old PLP‐hαSyn mice were fed over a period of 4 months with pellets containing anle138b at two different doses (0.6 and 2 g/kg) and compared to healthy controls and PLP‐hαSyn mice fed with placebo pellets. At the end of the treatment, behavioral and histological analyses were performed.

**Results:**

We observed a reversal of motor function to healthy control levels when PLP‐hαSyn mice were treated with both doses of anle138b. Histological and molecular analyses showed a significant reduction in α‐synuclein oligomers and glial cytoplasmic inclusions in animals fed with anle138b compared to nontreated mice. These animals also present preservation of dopaminergic neurons and reduction in microglial activation in SN correlating with the α‐synuclein reduction observed.

**Conclusions:**

Anle138b reduces α‐synuclein accumulation in PLP‐hαSyn mice, leading to neuroprotection, reduction of microglial activation, and preservation of motor function supporting the use of anle138b in a future clinical trial for MSA. © 2018 The Authors. *Movement Disorders* published by Wiley Periodicals, Inc. on behalf of International Parkinson and Movement Disorder Society.

Multiple system atrophy (MSA) is a rare and progressive neurodegenerative disorder characterized by autonomic failure and severe motor impairment leading to death a few years after symptom onset.^1^ No treatment to stop or reduce disease progression is available; only mitigation of some clinical symptoms may be achieved.^2^ MSA, together with Parkinson's disease (PD) and dementia with Lewy bodies, constitutes the family of synucleinopathies, characterized by misfolding and accumulation of α‐synuclein (α‐syn).^3^, ^4^, ^5^ In MSA, α‐syn accumulates in the cytoplasm of oligodendrocytes, forming the so‐called glial cytoplasmic inclusions (GCIs), thus differing from PD where α‐syn mainly accumulates in neurons in Lewy bodies.^3^, ^4^, ^5^ α‐syn accumulation in MSA leads to glial and neuronal dysfunction, neuroinflammation and finally neurodegeneration.^1^ MSA is divided in two different subtypes depending on the main areas affected by neurodegeneration. The Parkinson's variant or MSA‐P is characterized by striatonigral degeneration (SND), whereas the cerebellar variant or MSA‐C reflects olivopontocerebellar atrophy (OPCA).^1^ The PLP‐hαSyn transgenic mouse model recapitulates most of the clinical and pathophysiological features of MSA by overexpressing human α‐syn under the oligodendrocyte PLP (myelin proteolipid protein) promoter, which leads to GCI formation, microglial activation, and selective neurodegeneration.^6^, ^7^, ^8^, ^9^, ^10^, ^11^, ^12^, ^13^, ^14^, ^15^, ^16^

Microglial activation and neuroinflammation constitute important pathological features of MSA.^17^, ^18^, ^19^ Similarly to the human pathology, PLP‐hαSyn mice develop progressive microglial activation, initially triggered by α‐syn pathology in a region‐specific manner.^13^, ^15^ In a recent publication from our group, significant microglial activation was observed in the SN of MSA transgenic mice compared to healthy control animals at 5 months of age, and this activation was linked to abnormal neuroinflammatory response.^15^ PLP‐hαSyn mice also develop progressive SND characterized by a 30% reduction in the number of dopaminergic neurons (tyrosine hydroxylase positive [TH^+^] neurons) in the SNc compared to healthy control animals. This neuronal loss is already present at 4 months of age and is followed by a significant reduction in density of dopaminergic terminals and in number of medium spiny neurons in the striatum at 12 months of age.^15^, ^20^ SN and striatum are both essential for motor control, and the loss of neurons in these two brain areas leads to motor impairment in these mice.^12^, ^15^

According to several studies, under physiological conditions α‐syn is mainly located in the pre‐synaptic terminals of the neurons predominantly as a monomer.^21^ Although its function is still unknown, a possible role in neurotransmitter release, synaptic function, and plasticity has been suggested.^22^, ^23^ Misfolding, oligomerization, and aggregation of α‐syn are crucial events in the pathophysiology of synucleinopathies.^24^ In MSA, the origin of α‐syn inclusions in oligodendrocytes is still unknown and under discussion given that it is not clear whether or not mature oligodendrocytes express α‐syn.^25^, ^26^, ^27^ In the last years, several publications have shown the ability of α‐syn to be transferred from cell to cell and spread through brain parenchyma in a prion‐like manner.^28^, ^29^, ^30^, ^31^, ^32^, ^33^ Based on this, possible neuron‐oligodendrocyte transfer of α‐syn has been suggested in addition to the controversial oligodendrocytic origin of the misfolded α‐syn.^34^ Several studies in the last few years have shown that α‐syn oligomers constitute the main neurotoxic species for disease progression instead of large fibrillar deposits or inclusions.^35^, ^36^, ^37^, ^38^, ^39^ According to all these studies, the inhibition of α‐syn oligomerization constitutes a promising approach to fight the spreading of synucleinopathies and an important effort has been made in this direction.^21^, ^40^, ^41^ The use of small molecules to target α‐syn oligomerization and aggregation has shown promising results in preclinical models of PD as is the case of anle138b, a small compound with high bioavailability and low toxicity.^42^ This compound can be delivered orally and penetrates the blood–brain barrier, entering the brain with high efficacy.^42^ Thus, administration of anle138b as a food additive results in adequate and stable drug exposure.^43^ Anle138b has been shown effective in reducing disease progression in models of PD, prion disease, tauopathy, and Alzheimer's disease (AD) by inhibiting protein aggregation.^42^, ^43^, ^44^, ^45^ Based on this, we hypothesized that it could also be of use to attenuate disease progression in MSA. In order to test this hypothesis, we fed PLP‐hαSyn transgenic MSA mice with food pellets containing two different doses of anle138b followed by behavioral and histological analyses at the end of the treatment.

## Material and Methods

### Animals and Treatments

PLP‐hαSyn mice overexpressing wild‐type (WT) human α‐syn under the PLP promoter, an oligodendroglial‐specific promoter, and generated in a C57/BL6 background^16^ were used in this study. Animals were kept under temperature‐controlled, pathogen‐free conditions on a light/dark 12‐hour cycle. All the experiments were performed according to the ethical guidelines with the permission of the Austrian Federal Ministry of Science and Research (permission BMFWF‐66.011/0141‐WF/v/3b/2016). Two‐month‐old male transgenic mice were randomized in three different groups: one fed with placebo food pellets (n = 10; ssniff Spezialdiäten GmbH, Soest, Germany), another fed with pellets containing anle138b at 0.6 g/kg of food (n = 8; ssniff Spezialdiäten GmbH), and a last group fed with pellets containing 2 g of anle138b per kg of food (n = 10; ssniff Spezialdiäten GmbH). The dose of 2 g of anle138b per kg of food was used in previous experiments in mice^43^ and establishes during the wake phase a concentration of 60 μM in the brain. Two‐month‐old C57/BL6 healthy nontransgenic animals (WT) fed with placebo pellets were used as a healthy control (n = 10). Food pellets were provided to the animals throughout the whole experiment. After 4 months of treatment, behavioral analyses were performed followed by sacrifice of the animals and brain extraction.

### Behavioral Tests

#### *Challenging Beam Test*

At the end of the treatment, animal cages were randomly numbered by the animal caretaker and behavioral analyses were performed by the researcher blinded to genotype and treatment. Motor performance and coordination were analyzed with a modified version of the traditional beam test adapted from a previously published method.^46^, ^47^ Five performances were video recorded per animal, and the number of slips per step with the hind limbs was measured. The best three performances were used for statistical analyses.

### Tissue Processing and Histology

After behavioral analyses were performed, animals were perfused intracardially with phosphate buffered saline (PBS [pH 7.4]; Sigma‐Aldrich, St. Louis, MO) under deep thiopental anesthesia and brains were extracted. For molecular analyses, brains were snap frozen in liquid nitrogen and stored at –80 °C. For histological analyses, brains were postfixed overnight in 4% paraformaldehyde (pH 7.4; Sigma‐Aldrich) at 4 °C. After fixation, brains were washed in PBS and then transferred to 30% sucrose (in PBS) until they sank. Finally, brains were frozen using 2‐methylbutan (Sigma‐Aldrich) and stored at –80 °C for further analyses. In order to perform histological analyses, brains were serially cut in 40‐μm‐thick coronal sections using a freezing microtome (Leica Microsystems, Wetzlar, Germany) and stored free‐floating in a cryoprotectant buffer at –20 °C. One series was directly mounted on slides and stained with cresyl violet.

### Immunohistological Analyses

Free‐floating sections were stained following standard protocols. To analyze the number of GCIs, representative sections, including striatum and SN, were stained with the following antibodies: rat antihuman α‐syn 15G7 (1:200; Enzo Life Sciences, Farmingdale, NY) and mouse antioligomeric human α‐syn 5G4 (1:1,000; LINARIS GmbH, Mannheim, Germany). To analyze the number of dopaminergic neurons (TH^+^ neurons) in the SNc, serial sections were stained with rabbit anti‐TH antibody (1:1,000; Millipore, Burlington, MA). For microglial activation analysis, representative sections of SN were stained with rat anti‐CD68 antibody (1:200; R&D Systems, Minneapolis, MN). Sections were then incubated with biotinylated secondary antibodies, followed by Vectastain ABC reagent (Vector Laboratories, Burlingame, CA) and 3,3′‐diaminobenzidine (Sigma‐Aldrich), to visualize the immunohistochemical binding sites. Stained sections were mounted on slides, dehydrated, and coverslipped with Entellan (Merck & Co., Merck Kenilworth, NJ). For immunofluorescence, suitable immunoglobulin Gs, conjugated with Alexa 488 or Alexa 594 (Life Technologies, Carlsbad, CA), were applied, followed by nuclear staining with 4′,6‐diamidino‐2‐phenylindole (1:1,000; Sigma‐Aldrich) and finally coverslipped with mounting medium Fluromount‐G (SouthernBiotech, Birmingham, AL).

### Image Analyses

After immunostaining, and preceding the acquisition of images/stereological counting, all immunofluorescence/immunohistochemistry slides were randomly numbered by the laboratory technician. All measurements were therefore performed by the researcher blinded to genotype and treatment. Neuroanatomy was assessed using a Mouse Brain Atlas. Stereological analysis was performed using the Nikon E‐800 microscope equipped with a Nikon digital camera DXM1200 (Nikon, Tokyo, Japan) and Stereoinvestigator software (Microbrightfield Europe E.k., Magdeburg, Germany) as described previously.^48^ GCI density was assessed by meander scan throughout the area of interest and expressed in number of GCI/mm^2^. The number of TH^+^ and cresyl violet positive (CV^+^) neurons in the SNc was measured by applying the optical fractionator workflow. For microglial activation assessment, images were acquired with a fluorescence microscope (Leica DMI4000; Leica Microsystems), and the CD68‐positive area was estimated using ImageJ software (National Institutes of Health, Bethesda, MD). Results are presented as percentage of CD68 area per SN area.

### Continuous Sucrose‐Gradient Assay

For continuous gradient centrifugation, a 10% (w/v) brain homogenate of the midbrain was prepared using a buffer composed of 50 mM of Tris (pH 7.4), 175 mM of NaCl, 1 mM of MgCl_2_, 0.1 mM of phenylmethylsulfonyl fluoride, 1 mM of N‐ethylmaleimide, 0.1% Nonidet P‐40 Substitute, ethylenediaminetetraacetic acid–free cOmplete Mini protease inhibitor (Roche, Indianapolis, IN), and PhosSTOP phosphatase inhibitor (Roche). Aliquots of the homogenate were frozen in liquid N_2_ and stored at –80 °C. Total protein concentration was determined by bicinchoninic acid assay. For the sucrose gradient analysis, 300 μg of protein in a final volume of 200 μL were used. To this end, the brain homogenate was thawed on ice and diluted in a buffer containing 50 mM of Tris (pH 7.4), 175 mM of NaCl, 0.1% N‐lauroylsarcosine sodium salt (sarcosyl), and 0.5% sodium deoxycholate. Samples were agitated at 1,200 rpm (ThermoMixer C, Eppendorf) at 4 °C for 30 minutes. Cellular debris was removed afterward by centrifugation for 1 minute at 16.000 *g* and 4 °C. Sucrose gradients were prepared in a 4‐mL 11 × 60 mm polyallomer tube (Beckman Coulter, Brea, CA). To this end, sucrose solutions containing 50 mM of Tris (pH 7.4), 0.1% sarcosyl, and 10%, 20%, 30%, 40%, 50%, or 60% of sucrose were pipetted into the tube, starting with 200 μL of the 60% sucrose solution and followed by 400 μL of the 50% to 10% sucrose solutions. Two hundred microliters of the centrifugation supernatant was pipetted as the uppermost layer onto the gradient. Samples were ultracentrifuged in a SW 60 Ti rotor (Beckman Coulter) at 40,000 rpm and 4 °C for 1 hour. Twelve fractions of 200 μL each were collected from the top to the bottom of each tube and subjected to trichloroacetic acid (TCA) precipitation (10% TCA) overnight at –20 °C. After thawing, samples were centrifuged at 25,000 g and 4 °C for 15 minutes. Precipitates were washed once with acetone (–20 °C), centrifuged, and finally resuspended in Laemmli sample buffer. Samples were boiled at 96 °C for 5 minutes and subjected to sodium dodecyl sulfate polyacrylamide gel electrophoresis and western blotting. Antibodies against total α‐syn (4B12; BioLegend, San Diego, CA) and phosphorylated α‐syn (pS129; Abcam, Cambridge, MA) were used to stain the blots. Images were acquired using the Fusion FX system for western blot and gel imaging, and quantified with FUSION CAPT software (V16.09b; Vilber Lourmat Sté, Collégien, France).

### Statistical Analyses

All statistical analyses were conducted using the software Graph‐Pad Prism (version 7; GraphPad Software Inc., La Jolla, CA). Mean ± standard error of the mean (SEM) was used to present the results. One‐way analysis of variance (ANOVA) with a post‐hoc Bonferroni test was used to compare groups if not indicated otherwise. A *P* value <0.05 was considered statistically significant. Correlations were studied using linear regression analysis.

## Results

### Anle138b Prevents Motor Deficits and Neurodegeneration in the PLP‐hαSyn Mice

**Figure 1:**
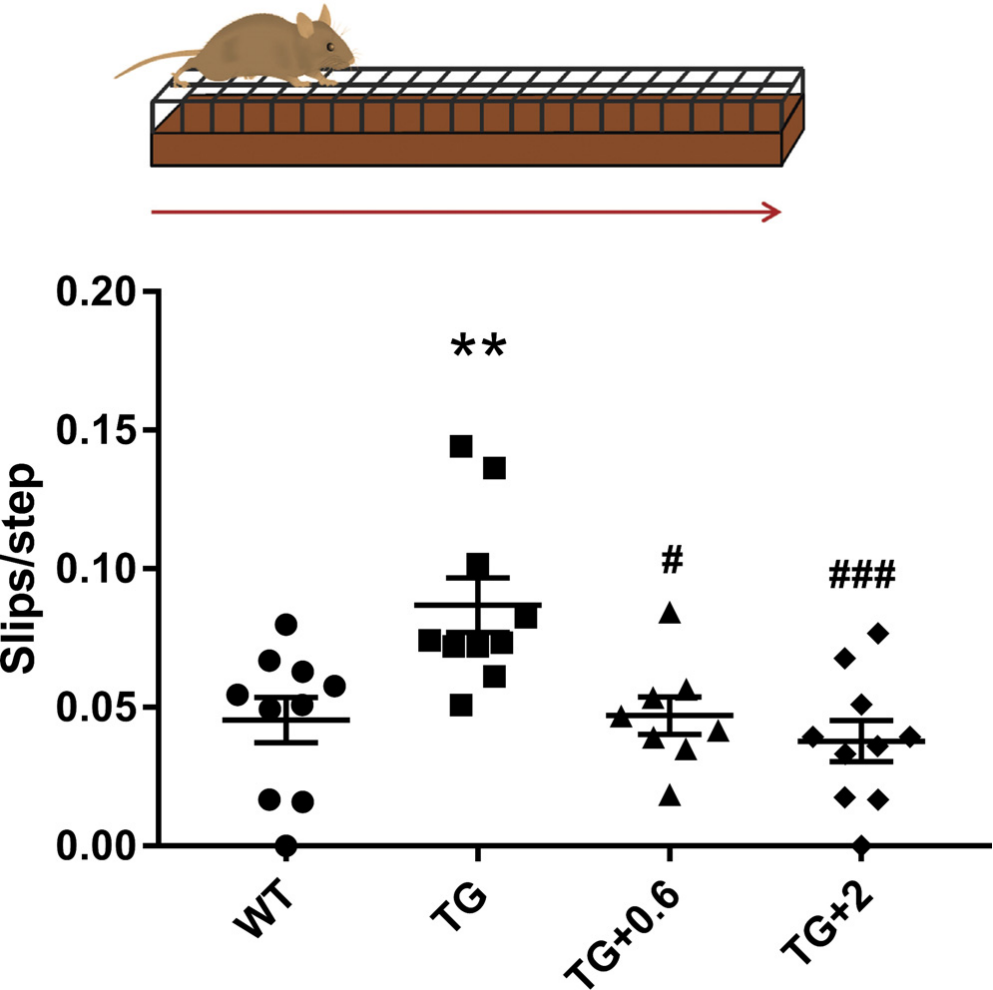
Anle138b treatment prevents motor impairment in PLP‐hαSyn mice. Schematic representation of the beam challenge test used for behavioral analysis. The number of slips when the animals traverse the beam were counted and normalized per the number of steps. n = 8 to 10 per experimental group. Error bars indicate SEM. ANOVA, slips per step/genotype: ^**^ *P* < 0.01; Slips per step/treatment: ^#^ *P* < 0.05; ^###^ *P* < 0.001 (Bonferroni's test). WT, wild‐type healthy control animals; TG, PLP‐hαSyn mice feed with placebo food pellets; TG + 0.6, PLP‐hαSyn mice feed with pellets containing anle138b at 0.6 g/kg of food; TG + 2, PLP‐hαSyn mice feed with pellets containing anle138b at 2 g/kg of food. [Color figure can be viewed at wileyonlinelibrary.com]

To assess the effect of anle138b on motor function, motor coordination and balance were analyzed using the beam challenging test^46^, ^47^ (Fig. 1). PLP‐hαSyn mice fed with placebo pellets showed a significant increase in the number of slips per step when traversing the beam compared to healthy control animals (Fig. 1). However, PLP‐hαSyn mice treated with both doses of anle138b maintained normal motor function as compared to healthy control mice (Fig. 1).

**Figure 2:**
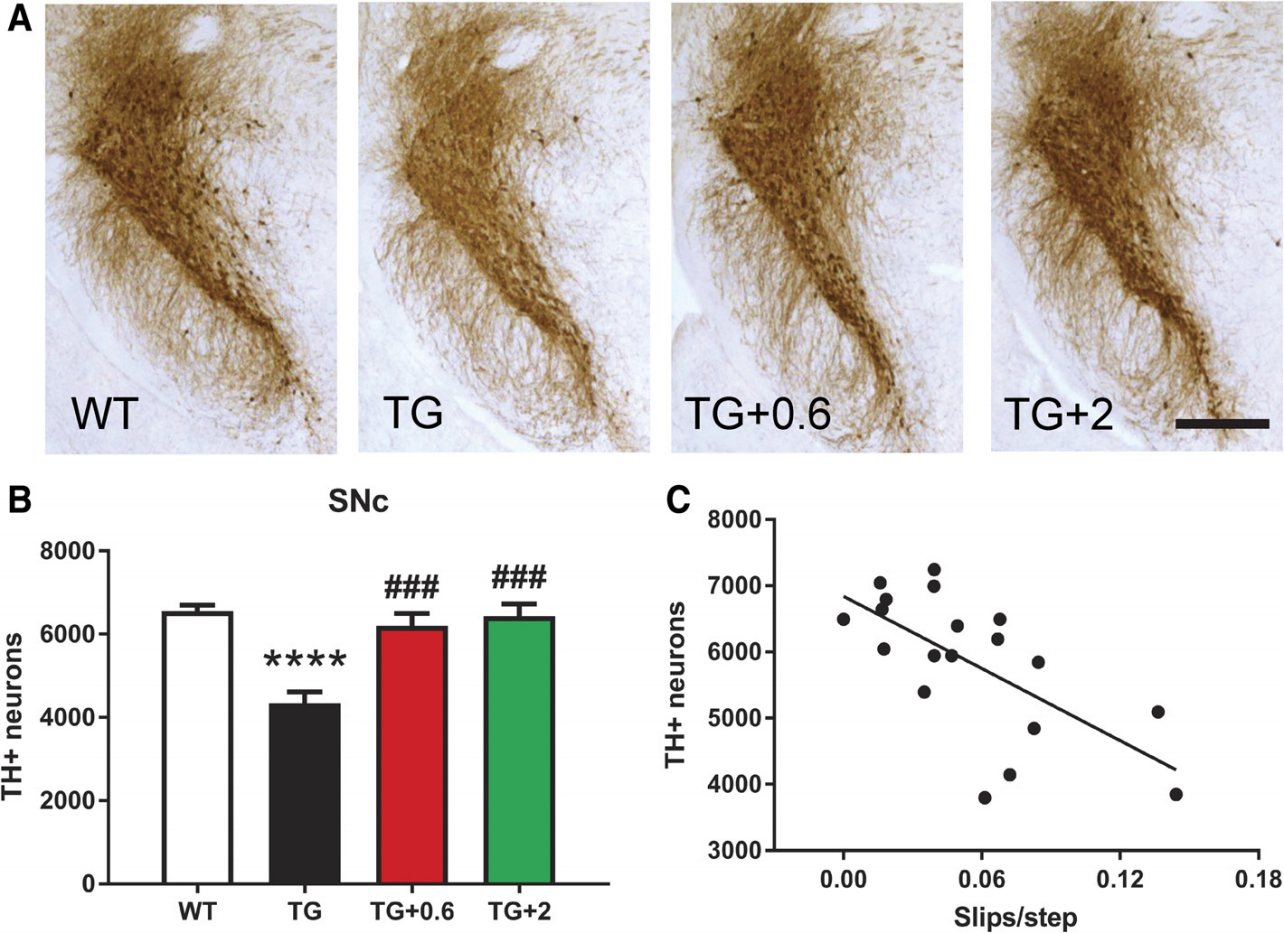
Anle138b prevents dopaminergic neuronal loss in the SNc of PLP‐hαSyn mice. (A) Representative images of SN sections stained against TH. Scale bar, 400 μm. (B) Stereological counting of the total number of dopaminergic (TH^+^) neurons in the entire SNc in the different groups. n = 4 to 5 per experimental group. Error bars indicate SEM. ANOVA, TH^+^ neurons/genotype: ^****^ *P* < 0.0001; TH^+^ neurons/treatment: ^###^ *P* < 0.001 (Bonferroni's test). (C), Correlation analysis of number of TH^+^ neurons in the SNc and number of slips per step. *P* = 0.0021; R^2^ = 0.4349. [Color figure can be viewed at wileyonlinelibrary.com]

To evaluate whether the improvement in motor function was related to a neuroprotective effect of anle138b on dopaminergic neurons in the SNc, the number of TH^+^ cells was quantified in this region. Stereological counting showed a clear preservation of dopaminergic neurons in the SNc of PLP‐hαSyn mice treated with both doses of anle138b compared to healthy controls (Fig. 2A,B). PLP‐hαSyn mice fed with placebo pellets, however, showed a significant loss of TH^+^ neurons compared to all groups (Fig. 2A,B). The neuroprotective effect of anle138b in SNc was validated by cresyl violet staining followed by stereological counting of neurons in this brain area (Supporting Information Fig. S1A). There was a significant correlation between the neuroprotective effect of anle138b and improvement in motor function (Fig. 2C and Supporting Information Fig. S1B).

### Anle138b Reduces α‐syn Accumulation in PLP‐hαSyn Mice

**Figure 3:**
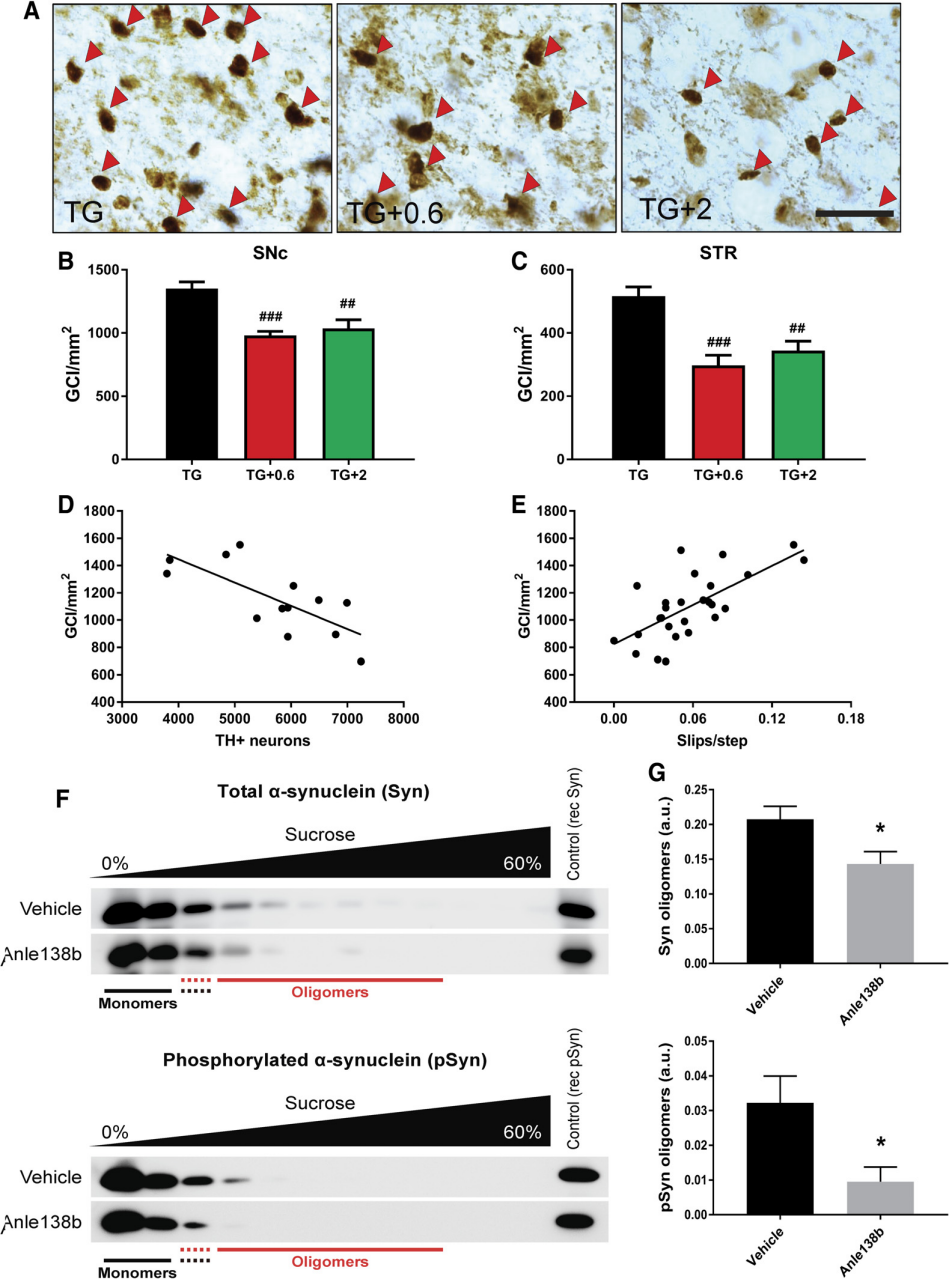
Anle138b reduces α‐syn accumulation in PLP‐hαSyn mice (A) Representative images of striatal sections stained with the antibody 15G7 against human α‐syn. Red arrows indicate individual GCI. Scale bar, 25 μm. (B,C) GCI density in SNc and STR (striatum) of PLP‐hαSyn mice determined by stereological counting of brain sections stained with 15G7 and expressed in GCI/mm^2^. n = 8 to 10 per experimental group. Error bars indicate SEM. ANOVA, GCI density/treatment: ^##^ *P* < 0.01; ^###^ *P* < 0.001 (Bonferroni's test). (D) Correlation analysis between the density of GCIs in the SNc and the number of TH^+^ neurons in the same region. *P* = 0.0041; R^2^ = 0.5415. (E) Correlation analysis of density of GCIs in the SNc and number of slips per step. *P* = 0.0001; R^2^ = 0.4546. (F) Representative blots of midbrain samples obtained after sucrose‐gradient centrifugation. Total α‐syn (upper panel) and phosphorylated α‐syn (lower panel) levels were analyzed in the different fractions. (G) Quantification of total α‐syn (upper panel) and phosphorylated α‐syn (lower panel) levels in the oligomeric fractions. n = 4 per experiental group. Error bars indicate SEM. Groups were compared by unpaired two‐tailed *t* test. ^*^ *P* < 0.05. [Color figure can be viewed at wileyonlinelibrary.com]

Next, the effect of anle138b on α‐syn aggregation and intracellular accumulation was assessed. Brain sections of PLP‐hαSyn mice were immunostained by using antibodies against α‐syn, and the number of GCIs per area in striatum and SN was analyzed. Both doses of anle138b showed a significant effect on GCI number in SNc and striatum, where a 30% reduction was observed (Fig. 3A–C and Supporting Information Fig. S2A,B). This effect was confirmed with the different antibodies used in the study (Fig. 3A–C and Supporting Information Fig. S2A,B). There was a significant correlation between GCI reduction and preservation of neurons in SNc (Fig. 3D and Supporting Information Fig. S1C,D and S2C) as well as motor function (Fig. 3E and Supporting Information Fig. S2D).

In addition to the effect on the amount of GCIs, sucrose‐gradient analysis showed a significant reduction of α‐syn oligomers in midbrains of PLP‐hαSyn mice treated with anle138b (Fig. 3F,G). This reduction was observed not only in total α‐syn oligomers, but also in pathological phosphorylated α‐syn oligomers (Fig. 3F,G), thus confirming the modulatory effect of the small molecule on α‐syn oligomerization.

### Anle138b Reduces Microglial Activation in PLP‐hαSyn Mice

**Figure 4:**
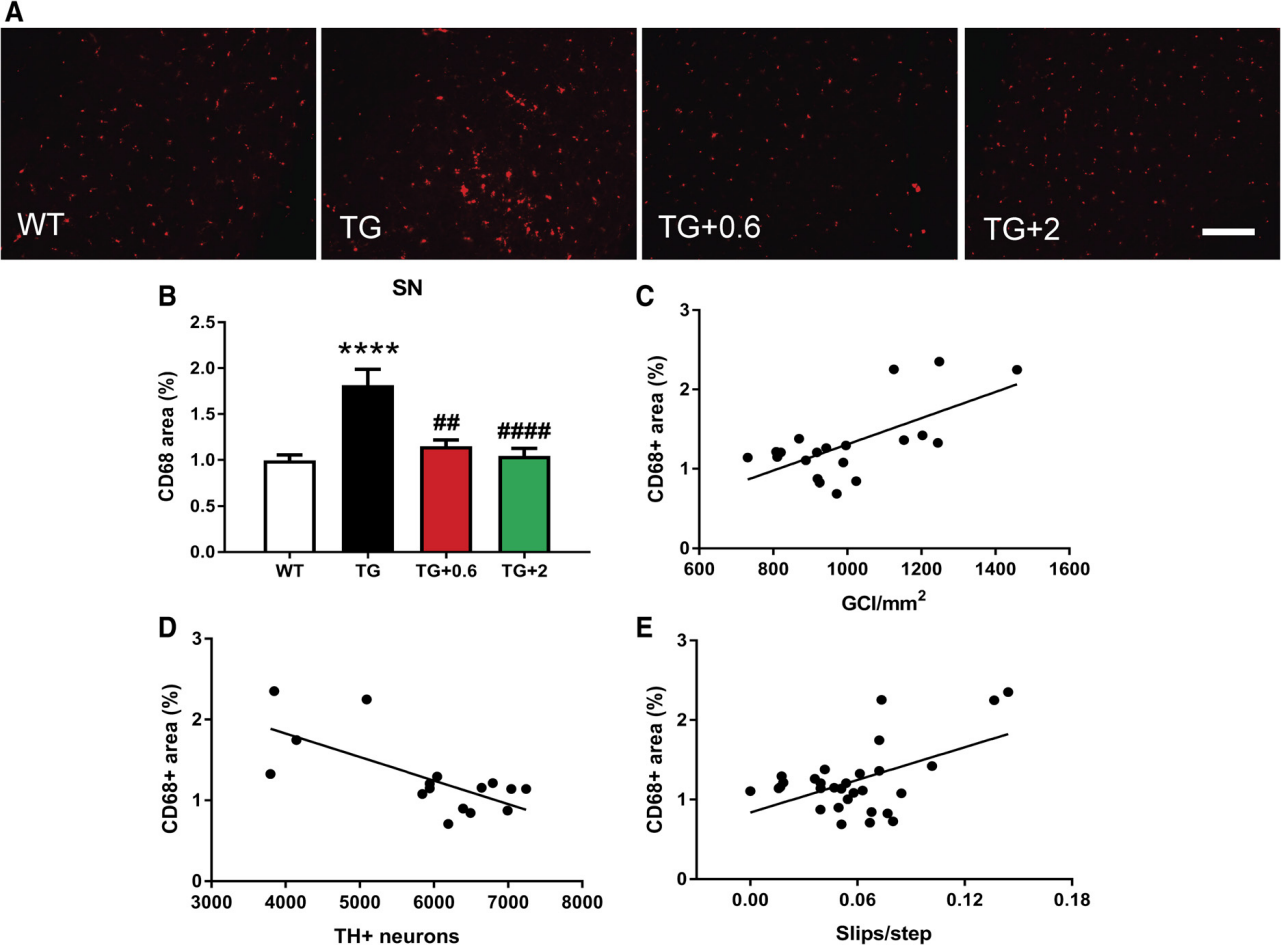
Anle138b reduces microglial activation in PLP‐hαSyn mice. (A) Representative immunofluorescence images of SN sections stained against CD68. Scale bar, 150 μm. (B) CD68‐positive (CD68^+^) area in the different groups, determined by ImageJ analysis (National Institutes of Health, Bethesda, MD) and expressed as the % of the total area of the SN. n = 6 to 9 per experimental group. Error bars indicate SEM. ANOVA, CD68^+^ area/genotype: ^****^ *P* < 0.0001; CD68^+^ area/treatment: ^##^ *P* < 0.01; ^####^ *P* < 0.0001 (Bonferroni's test). (C) Correlation analysis of CD68^+^ area in the SN and density of GCIs (15G7) in the same brain region. *P* = 0.0017; R^2^ = 0.4288. (D) Correlation analysis between the CD68^+^ area in the SN and the number of TH^+^ neurons in the SNc. *P* = 0.0027; R^2^ = 0.4862. (E) Correlation analysis of CD68^+^ area in the SN and number of slips per step. *P* = 0.0033; R^2^ = 0.2689. [Color figure can be viewed at wileyonlinelibrary.com]

To assess the effect of anle138b treatment on microglial activation, we performed immunofluorescence microscopy for CD68, a lysosomal marker indicative of phagocytic activity of microglia^49^ associated with α‐syn accumulation.^15^, ^50^, ^51^, ^52^ In agreement with previous results,^15^ significant microglial activation was observed at this stage in the SN of the PLP‐hαSyn placebo group compared to healthy control animals (Fig. 4A,B). In contrast, both doses of anle138b significantly reduced microglial activation in the SN back to its normal levels as observed in healthy control mice (Fig. 4A,B). There was a significant correlation between the reduction of microglial activation in SN, GCI reduction (Fig. 4C and Supporting Information Fig. S2E), preservation of neurons (Fig. 4D and Supporting Information Fig. S1E), as well as motor function (Fig. 4E).

## Discussion

Aggregation and accumulation of misfolded proteins constitute a key factor in the progression of several neurodegenerative diseases. Thus, the use of compounds or therapies capable of reducing or blocking abnormal protein aggregation represents a promising strategy to attenuate the clinical progression in these disorders. Synucleinopathies are pathologically characterized by the abnormal aggregation and accumulation of α‐syn, and, in recent years, different approaches have been developed to target this process.^3^, ^4^, ^5^, ^21^, ^40^, ^41^ The use of small molecules and immunotherapy constitute two of the main strategies with therapeutic potential according to preclinical in vivo and in vitro models.^53^, ^54^ Small molecules present important advantages such as their high oral bioavailability and blood–brain barrier penetration and their ability to act inside cells.^53^

Here we show that anle138b, which targets specifically oligomers, reduces α‐syn accumulation in a mouse model of early‐stage MSA, thus corroborating the oligomer modulation effect on α‐syn previously observed in PD models and the general effect observed also in Abeta, tau, and prion aggregation mouse models.^42^, ^43^, ^44^, ^45^ An important advantage of this small molecule, from a therapeutic point of view, is that it does not bind to α‐syn monomers, therefore preserving its physiological functions.^42^ At the molecular level, anle138b blocks the formation of toxic α‐syn oligomers,^42^ which, in pathological conditions, can induce neuronal damage through the formation of pores in the cell membrane and mitochondria^21^, ^39^, ^55^, ^56^ and trigger the activation of microglial cells.^52^, ^57^, ^58^, ^59^ Because formation of α‐syn oligomers is blocked, also less α‐syn fibrils are generated. In accord with this, our data show that oral administration of anle138b reduces α‐syn oligomers in midbrains of PLP‐hαSyn mice and leads to a 30% reduction in the number of GCI in the striatum and SN of MSA mice, two brain areas associated with motor function. We also demonstrate that the reduction of α‐syn accumulation prevents loss of dopaminergic nigral neurons and motor impairment, thus confirming the therapeutic effect of anle138b observed also in PD models.^42^, ^44^ Finally, our data show that the decrease of α‐syn aggregation in the SN is associated with a reduction of microglial activation back to the levels observed in healthy control animals in this brain region, which also correlates with the neuroprotective effect of anle138b on dopaminergic cells and preservation of motor function.

The results presented here demonstrate that anle138b treatment is beneficial in early‐stage MSA as modeled by young PLP‐hαSyn mice.^15^ However, efficacy of anle138b was less prominent in a model of pathologically advanced MSA.^60^ In that study, the potential of anle138b was evaluated in 1‐year‐old PLP‐hαSyn mice treated with the mitochondrial toxin, 3‐nitropropionic acid (3‐NP), to trigger full‐blown MSA‐like pathology, with spreading of GCI, SND, and OPCA and strong microglial activation.^12^ Behavioral analyses showed significant motor improvement after short‐term treatment with anle138b; however, no significant changes in α‐syn aggregate load or cell death were observed in these animals.^60^ The variable efficacy of anle138b in the two studies may be attributed to short (1 month) versus long‐term (4 months) treatment, but also reflect the fact that PLP‐hαSyn animals had sustained different degrees of neurodegeneration in the absence or presence of 3‐NP exposure. Moreover, the limited efficacy of anle138b in our first experiment may have been attributed to the addition of toxin‐induced acute oxidative stress that cannot be counteracted by the administration of anle138b, given that it has no antioxidant properties. In contrast, to clarify the antiaggregation and neuroprotective potential of anle138b, a different, preventive approach was used in the present study, where chronic administration of the compound started before nigral degeneration of dopaminergic neurons occurred and in the absence of other non‐αSyn‐dependent deleterious stimuli.

**Figure 5:**
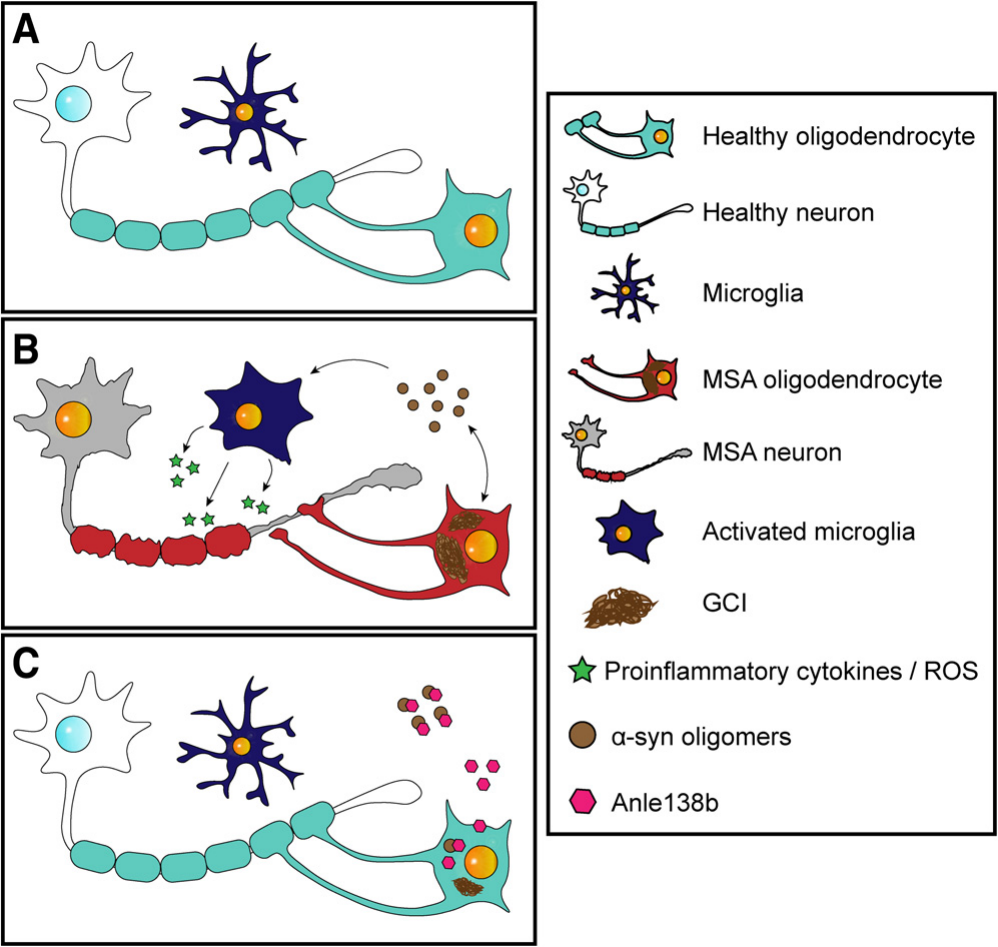
Pathophysiological features of MSA and potential therapeutic effect of anle138b. (A) Schematic overview of the CNS in healthy conditions. (B) During MSA pathogenesis α‐syn accumulates in the cytoplasm of oligodendrocytes inducing oligodendroglial dysfunction. Toxic oligomeric species of α‐syn are also formed and spread through the brain parenchyma triggering microglial activation and neuroinflammation. All these events lead finally to demyelination and neurodegeneration. (C) Treatment with anle138b would reduce the formation of GCIs and other toxic α‐syn species and would attenuate neuroinflammation, demyelination, and neurodegeneration in MSA patients. [Color figure can be viewed at wileyonlinelibrary.com]

In summary, our study shows that oral administration of anle138b with initiation in the early disease stages constitutes a promising approach to prevent disease progression in MSA (Fig. 5). Our observations and previous studies have demonstrated the potential of anle138b in different neurodegenerative disorders, and the data presented here support further development of anle138b for future clinical applications in patients suffering from MSA or related synucleinopathies.

## Author Roles

(1) Research Project: A. Conception and Design; B. Acquisition of Data; C. Analysis and Interpretation of Data; (2) Manuscript: A. Writing of the First Draft, B. Review and Critique; (3) Other: A. Final Approval of the Submission Statistical Analysis; B. Obtaining Funding; C. Technical Support; D. Supervision of Data Collection.

A.H.G.: 1B, 1C, 2A, 2B, 3A.

D.W.: 1B, 1C, 2A, 2B, 3A.

S.R.: 1C, 2B, 3A.

A.L.: 1C, 2B, 3A.

C.G.: 1A, 1C, 2B, 3A, 3D.

A.G.: 1A, 1C, 2B, 3A, 3D.

G.W.: 1A, 1C, 2B, 3A, 3B, 3D.

N.S.: 1A, 1C, 2B, 3A, 3B, 3D.

## Financial Disclosures

A.H.‐G. has been employed by Medical University of Innsbruck. D.W. has been employed by MODAG GmbH. S.R. holds intellectual property rights with EP patent application 17170855 and has been employed by Max Planck Institute for Biophysical Chemistry. A.L. holds intellectual property rights with

EP Patent Application 17170855 and has been employed by Max Planck Institute for Biophysical Chemistry and MODAG GmbH. C.G. holds stock ownership in medically related fields: AMGEN, Bayer, Eli Lilly, Fresenius Healthcare, GlaxoSmithKline, Medtronic, Merck, Novartis, Pfizer, Procer Gamble, and Sanofi; patents of the last 12 months: compounds for the treatment and prevention of melanoma: PCT/EP2018/062236; consultancies: MODAG GmbH, Wendelsheim, Germany; expert testimony: ERC, Norwegian Science Foundation; advisory boards: TIFR, uNMR‐NL, and TGIR RMN THC; has been employed by Max Planck Society; Partnerships: KBSI, Daejon, Korea and MPLbioR, Rosario, Argentina; honoraria: Sanofi Aventis: 200€ for a lecture (2018); and Grants: DFG, FP7 health, and BMWi. A.G. stock ownership in medically related fields: shareholder of MODAG GmbH; intellectual property rights: several patents on anle138b and related compounds; employment: Ludwig‐Maximilians‐University Munich; and grants: Michael J Fox Foundation, FP7 health, BMBF. G.K.W. consultancies: Astra; advisory boards: Biogen; employment: Medical University of Innsbruck; honoraria: Novartis, Prana, Lundbeck, Affiris, and AbbVie; royalties: Springer, Cambridge University Press; grants: FWF, FP7 health, and MSA Coalition. N.S. has been employed by Medical University of Innsbruck and has received grants: FWF, FP7 health, and MSA Coalition.

## Supporting information

Supporting Information FIG. S1. Anle138b prevents neuronal loss in the SNc of PLP‐hαSyn mice. (A) Stereological counting of the total number of cresyl violet positive (CV^+^) neurons in SNc . n = 5 per experimental group. Error bars indicate SEM. ANOVA, CV^+^ neurons/genotype: ^****^ *P* < 0.0001; CV^+^ neurons/treatment: ^####^ *P* < 0.0001 (Bonferroni's test). (B) Correlation analysis of number of neurons in the SNc and number of slips per step. *P* < 0.0001; R^2^ = 0.6478. (C,D) Correlation analysis of the number of CV^+^ neurons in the SNc and the density of GCIs (15G7 and 5G4 respectively) in the SNc*. P* = 0.0010, R^2^ = 0.6076 and *P* = 0.0079, R^2^ = 0.4884 respectively. (E) Correlation analysis of number of CV^+^ neurons in the SNc and CD68^+^ area in the SN. *P* = 0.0002; R^2^ = 0.6051.

Supporting Information FIG. 2. Anle138b reduces α‐syn accumulation and microglial activation in PLP‐hαSyn mice. (A,B) GCI density in SNc and STR of PLP‐hαSyn mice determined by stereological counting of brain sections stained with the 5G4 antibody against oligomeric human α‐syn and expressed in GCI/mm^2^. n = 8 to 9 per experimental group. Error bars indicate SEM. ANOVA, GCI density/treatment: ^##^ *P* < 0.01; ^###^ *P* < 0.001 (Bonferroni's test). (C) Correlation analysis of density of GCIs (5G4) in the SNc and number of TH^+^ neurons in the same region. *P* = 0.0368; R^2^ = 0.3672. (D) Correlation analysis of density of GCIs (5G4) in the SNc and number of slips per step. *P* = 0.0026; R^2^ = 0. 3194. (E) Correlation analysis between the CD68^+^ area in the SN and the density of GCIs (5G4) in the same brain region. *P* = 0. 0079; R^2^ = 0. 3477.

## References

[1] Fanciulli A , Wenning GK . Multiple‐system atrophy. N Engl J Med 2015;372:249–263. 25587949 10.1056/NEJMra1311488

[2] Eschlbock S , Krismer F , Wenning GK . Interventional trials in atypical parkinsonism. Parkinsonism Relat Disord 2016;22(Suppl 1):S82–S92. 26421389 10.1016/j.parkreldis.2015.09.038

[3] Spillantini MG , Schmidt ML , Lee VM , Trojanowski JQ , Jakes R , Goedert M . Alpha‐synuclein in Lewy bodies. Nature 1997;388:839–40. 9278044 10.1038/42166

[4] Trojanowski JQ , Lee VM **.** Parkinson's disease and related alpha‐synucleinopathies are brain amyloidoses. Ann N Y Acad Sci 2003;991:107–110. 12846979 10.1111/j.1749-6632.2003.tb07468.x

[5] Tu PH , Galvin JE , Baba M , et al. Glial cytoplasmic inclusions in white matter oligodendrocytes of multiple system atrophy brains contain insoluble alpha‐synuclein. Ann Neurol 1998;44:415–422. 9749615 10.1002/ana.410440324

[6] Boudes M , Uvin P , Pinto S , et al. Bladder dysfunction in a transgenic mouse model of multiple system atrophy. Mov Disord 2013;28:347–355. 23426727 10.1002/mds.25336 PMC4743066

[7] Fernagut PO , Meissner WG , Biran M , et al. Age‐related motor dysfunction and neuropathology in a transgenic mouse model of multiple system atrophy. Synapse. 2014;68:98–106. 24243499 10.1002/syn.21719

[8] Flabeau O , Meissner WG , Ozier A , Berger P , Tison F , Fernagut PO . Breathing variability and brainstem serotonergic loss in a genetic model of multiple system atrophy. Mov Disord 2014;29:388–395. 24442757 10.1002/mds.25804

[9] Hartner L , Keil TW , Kreuzer M , et al. Distinct parameters in the EEG of the PLP alpha‐SYN mouse model for multiple system atrophy reinforce face validity. Front Behav Neurosci 2016;10:252. 28119583 10.3389/fnbeh.2016.00252 PMC5222844

[10] Krismer F , Wenning GK , Li Y , Poewe W , Stefanova N . Intact olfaction in a mouse model of multiple system atrophy. PLoS One 2013;8:e64625. 23691255 10.1371/journal.pone.0064625 PMC3656866

[11] Kuzdas D , Stemberger S , Gaburro S , Stefanova N , Singewald N , Wenning GK . Oligodendroglial alpha‐synucleinopathy and MSA‐like cardiovascular autonomic failure: experimental evidence. Exp Neurol 2013;247:531–536. 23399889 10.1016/j.expneurol.2013.02.002 PMC3748345

[12] Stefanova N , Reindl M , Neumann M , et al. Oxidative stress in transgenic mice with oligodendroglial alpha‐synuclein overexpression replicates the characteristic neuropathology of multiple system atrophy. Am J Pathol 2005;166:869–876. 15743798 10.1016/s0002-9440(10)62307-3 PMC1602361

[13] Stefanova N , Reindl M , Neumann M , Kahle PJ , Poewe W , Wenning GK . Microglial activation mediates neurodegeneration related to oligodendroglial alpha‐synucleinopathy: implications for multiple system atrophy. Mov Disord 2007;22:2196–2203. 17853477 10.1002/mds.21671

[14] Stemberger S , Poewe W , Wenning GK , Stefanova N . Targeted overexpression of human alpha‐synuclein in oligodendroglia induces lesions linked to MSA‐like progressive autonomic failure. Exp Neurol 2010;224:459–464. 20493840 10.1016/j.expneurol.2010.05.008 PMC2913120

[15] Refolo V , Bez F , Polissidis A , et al. Progressive striatonigral degeneration in a transgenic mouse model of multiple system atrophy: translational implications for interventional therapies. Acta Neuropathol Commun 2018;6:2. doi: 10.1186/s40478-017-0504-y. 29298733 PMC5753576

[16] Kahle PJ , Neumann M , Ozmen L , et al. Hyperphosphorylation and insolubility of alpha‐synuclein in transgenic mouse oligodendrocytes. EMBO Rep 2002;3:583–588. 12034752 10.1093/embo-reports/kvf109 PMC1084143

[17] Gerhard A , Banati RB , Goerres GB , et al. [11C](R)‐PK11195 PET imaging of microglial activation in multiple system atrophy. Neurology 2003;61:686–689. 12963764 10.1212/01.wnl.0000078192.95645.e6

[18] Vieira BD , Radford RA , Chung RS , Guillemin GJ , Pountney DL . Neuroinflammation in multiple system atrophy: response to and cause of alpha‐synuclein aggregation. Front Cell Neurosci 2015;9:437. 26778958 10.3389/fncel.2015.00437 PMC4700780

[19] Ishizawa K , Komori T , Sasaki S , Arai N , Mizutani T , Hirose T . Microglial activation parallels system degeneration in multiple system atrophy. J Neuropathol Exp Neurol 2004;63:43–52. 14748560 10.1093/jnen/63.1.43

[20] Bassil F , Fernagut PO , Bezard E , et al. Reducing C‐terminal truncation mitigates synucleinopathy and neurodegeneration in a transgenic model of multiple system atrophy. Proc Natl Acad Sci U S A 2016;113:9593–9598. 27482103 10.1073/pnas.1609291113 PMC5003293

[21] Lashuel HA , Overk CR , Oueslati A , Masliah E . The many faces of alpha‐synuclein: from structure and toxicity to therapeutic target. Nat Rev Neurosci 2013;14:38–48. 23254192 10.1038/nrn3406 PMC4295774

[22] Bendor JT , Logan TP , Edwards RH . The function of alpha‐synuclein. Neuron 2013;79:1044–1066. 24050397 10.1016/j.neuron.2013.09.004 PMC3866954

[23] Burre J , Sharma M , Tsetsenis T , Buchman V , Etherton MR , Sudhof TC . Alpha‐synuclein promotes SNARE‐complex assembly in vivo and in vitro. Science 2010;329:1663–1667. 20798282 10.1126/science.1195227 PMC3235365

[24] Wong YC , Krainc D**.** alpha‐synuclein toxicity in neurodegeneration: mechanism and therapeutic strategies. Nat Med 2017;23:1–13. 10.1038/nm.4269 PMC8480197 28170377

[25] Asi YT , Simpson JE , Heath PR , et al. Alpha‐synuclein mRNA expression in oligodendrocytes in MSA. Glia 2014;62:964–970. 24590631 10.1002/glia.22653 PMC4238782

[26] Djelloul M , Holmqvist S , Boza‐Serrano A , et al. Alpha‐synuclein expression in the oligodendrocyte lineage: an in vitro and in vivo study using rodent and human models. Stem Cell Reports 2015;5:174–184. 26235891 10.1016/j.stemcr.2015.07.002 PMC4618831

[27] Miller DW , Johnson JM , Solano SM , Hollingsworth ZR , Standaert DG , Young AB . Absence of alpha‐synuclein mRNA expression in normal and multiple system atrophy oligodendroglia. J Neural Transm (Vienna) 2005;112:1613–1624. 16284907 10.1007/s00702-005-0378-1

[28] Kordower JH , Chu Y , Hauser RA , Freeman TB , Olanow CW . Lewy body‐like pathology in long‐term embryonic nigral transplants in Parkinson's disease. Nat Med 2008;14:504–506. 18391962 10.1038/nm1747

[29] Kovacs GG , Breydo L , Green R , Kis V , Puska G , Lorincz P , et al. Intracellular processing of disease‐associated alpha‐synuclein in the human brain suggests prion‐like cell‐to‐cell spread. Neurobiol Dis 2014;69:76–92. 24878508 10.1016/j.nbd.2014.05.020

[30] Recasens A , Dehay B , Bove J , et al. Lewy body extracts from Parkinson disease brains trigger alpha‐synuclein pathology and neurodegeneration in mice and monkeys. Ann Neurol 2014;75:351–362. 24243558 10.1002/ana.24066

[31] Luk KC , Kehm V , Carroll J , et al. Pathological alpha‐synuclein transmission initiates Parkinson‐like neurodegeneration in nontransgenic mice. Science 2012;338:949–953. 23161999 10.1126/science.1227157 PMC3552321

[32] Watts JC , Giles K , Oehler A , et al. Transmission of multiple system atrophy prions to transgenic mice. Proc Natl Acad Sci U S A 2013;110:19555–19560. 24218576 10.1073/pnas.1318268110 PMC3845125

[33] Peelaerts W , Bousset L , Van der Perren A , et al. alpha‐Synuclein strains cause distinct synucleinopathies after local and systemic administration. Nature 2015;522:340–344. 26061766 10.1038/nature14547

[34] Reyes JF , Rey NL , Bousset L , Melki R , Brundin P , Angot E . Alpha‐synuclein transfers from neurons to oligodendrocytes. Glia 2014;62:387–398. 24382629 10.1002/glia.22611

[35] Kalia LV , Kalia SK , McLean PJ , Lozano AM , Lang AE. alpha‐Synuclein oligomers and clinical implications for Parkinson disease. Ann Neurol 2013;73:155–169. 23225525 10.1002/ana.23746 PMC3608838

[36] Winner B , Jappelli R , Maji SK , et al. In vivo demonstration that alpha‐synuclein oligomers are toxic. Proc Natl Acad Sci U S A 2011;108:4194–4199. 21325059 10.1073/pnas.1100976108 PMC3053976

[37] Volles MJ , Lansbury PT , Jr. Zeroing in on the pathogenic form of alpha‐synuclein and its mechanism of neurotoxicity in Parkinson's disease. Biochemistry 2003;42:7871–7878. 12834338 10.1021/bi030086j

[38] Outeiro TF , Putcha P , Tetzlaff JE , Spoelgen R , Koker M , Carvalho F , et al. Formation of toxic oligomeric alpha‐synuclein species in living cells. PLoS One 2008;3:e1867. 18382657 10.1371/journal.pone.0001867 PMC2270899

[39] Kostka M , Hogen T , Danzer KM , et al. Single particle characterization of iron‐induced pore‐forming alpha‐synuclein oligomers. J Biol Chem 2008;283:10992–11003. 18258594 10.1074/jbc.M709634200

[40] Eisele YS , Monteiro C , Fearns C , et al. Targeting protein aggregation for the treatment of degenerative diseases. Nat Rev Drug Discov 2015;14:759–780. 26338154 10.1038/nrd4593 PMC4628595

[41] Gadad BS , Britton GB , Rao KS . Targeting oligomers in neurodegenerative disorders: lessons from alpha‐synuclein, tau, and amyloid‐beta peptide. J Alzheimers Dis 2011;24(Suppl 2):223–232. 21460436 10.3233/JAD-2011-110182

[42] Wagner J , Ryazanov S , Leonov A , et al. Anle138b: a novel oligomer modulator for disease‐modifying therapy of neurodegenerative diseases such as prion and Parkinson's disease. Acta Neuropathol 2013;125:795–813. 23604588 10.1007/s00401-013-1114-9 PMC3661926

[43] Wagner J , Krauss S , Shi S , et al. Reducing tau aggregates with anle138b delays disease progression in a mouse model of tauopathies. Acta Neuropathol 2015;130:619–631. 26439832 10.1007/s00401-015-1483-3 PMC4612332

[44] Levin J , Schmidt F , Boehm C , et al. The oligomer modulator anle138b inhibits disease progression in a Parkinson mouse model even with treatment started after disease onset. Acta Neuropathol 2014;127:779–780. 24615514 10.1007/s00401-014-1265-3 PMC3984662

[45] Martinez Hernandez A , Urbanke H , Gillman AL , et al. The diphenylpyrazole compound anle138b blocks Abeta channels and rescues disease phenotypes in a mouse model for amyloid pathology. EMBO Mol Med 2018;10:32–47. 29208638 10.15252/emmm.201707825 PMC5760857

[46] Fleming SM , Salcedo J , Fernagut PO , et al. Early and progressive sensorimotor anomalies in mice overexpressing wild‐type human alpha‐synuclein. J Neurosci 2004;24:9434–9440. 15496679 10.1523/JNEUROSCI.3080-04.2004 PMC6730110

[47] Fleming SM , Ekhator OR , Ghisays V . Assessment of sensorimotor function in mouse models of Parkinson's disease. J Vis Exp 2013;(76):50303. 10.3791/50303 PMC3727502 23851663

[48] Stefanova N , Kaufmann WA , Humpel C , Poewe W , Wenning GK . Systemic proteasome inhibition triggers neurodegeneration in a transgenic mouse model expressing human alpha‐synuclein under oligodendrocyte promoter: implications for multiple system atrophy. Acta Neuropathol 2012;124:51–65. 22491959 10.1007/s00401-012-0977-5 PMC3377902

[49] Zotova E , Bharambe V , Cheaveau M , et al. Inflammatory components in human Alzheimer's disease and after active amyloid‐beta42 immunization. Brain 2013;136(Pt 9):2677–2696. 23943781 10.1093/brain/awt210

[50] Doorn KJ , Moors T , Drukarch B , van de Berg W , Lucassen PJ , van Dam AM . Microglial phenotypes and toll‐like receptor 2 in the substantia nigra and hippocampus of incidental Lewy body disease cases and Parkinson's disease patients. Acta Neuropathol Commun 2014;2:90. 25099483 10.1186/s40478-014-0090-1 PMC4224021

[51] Theodore S , Cao S , McLean PJ , Standaert DG . Targeted overexpression of human alpha‐synuclein triggers microglial activation and an adaptive immune response in a mouse model of Parkinson disease. J Neuropathol Exp Neurol 2008;67:1149–1158. 19018246 10.1097/NEN.0b013e31818e5e99 PMC2753200

[52] Fellner L , Irschick R , Schanda K , et al. Toll‐like receptor 4 is required for alpha‐synuclein dependent activation of microglia and astroglia. Glia 2013;61:349–360. 23108585 10.1002/glia.22437 PMC3568908

[53] Guerrero‐Munoz MJ , Castillo‐Carranza DL , Kayed R . Therapeutic approaches against common structural features of toxic oligomers shared by multiple amyloidogenic proteins. Biochem Pharmacol 2014;88:468–478. 24406245 10.1016/j.bcp.2013.12.023

[54] Valera E , Spencer B , Masliah E . Immunotherapeutic approaches targeting amyloid‐beta, alpha‐synuclein, and tau for the treatment of neurodegenerative disorders. Neurotherapeutics 2016;13:179–189. 26494242 10.1007/s13311-015-0397-z PMC4720672

[55] Lashuel HA , Hartley D , Petre BM , Walz T , Lansbury PT, Jr . Neurodegenerative disease: amyloid pores from pathogenic mutations. Nature. 2002;418:291. 10.1038/418291a 12124613

[56] Lotharius J , Brundin P . Pathogenesis of Parkinson's disease: dopamine, vesicles and alpha‐synuclein. Nat Rev Neurosci 2002;3:932–942. 12461550 10.1038/nrn983

[57] Kim C , Ho DH , Suk JE , et al. Neuron‐released oligomeric alpha‐synuclein is an endogenous agonist of TLR2 for paracrine activation of microglia. Nat Commun 2013;4:1562. 23463005 10.1038/ncomms2534 PMC4089961

[58] Lee HJ , Bae EJ , Lee SJ . Extracellular alpha‐synuclein—a novel and crucial factor in Lewy body diseases. Nat Rev Neurol 2014;10:92–98. 24468877 10.1038/nrneurol.2013.275

[59] Zhang W , Wang T , Pei Z , et al. Aggregated alpha‐synuclein activates microglia: a process leading to disease progression in Parkinson's disease. FASEB J 2005;19:533–542. 15791003 10.1096/fj.04-2751com

[60] Fellner L , Kuzdas‐Wood D , Levin J , et al. Anle138b partly ameliorates motor deficits despite failure of neuroprotection in a model of advanced multiple system atrophy. Front Neurosci 2016;10:99. 27013960 10.3389/fnins.2016.00099 PMC4785146

