# Supplementary figures and images for: Anle138b modulates α‐synuclein oligomerization and prevents motor decline and neurodegeneration in a mouse model of multiple system atrophy
Source: Mov Disord. 2018 Nov 19;34(2):255–63. doi: 10.1002/mds.27562 (PMC6492169; doi:10.1002/mds.27562)

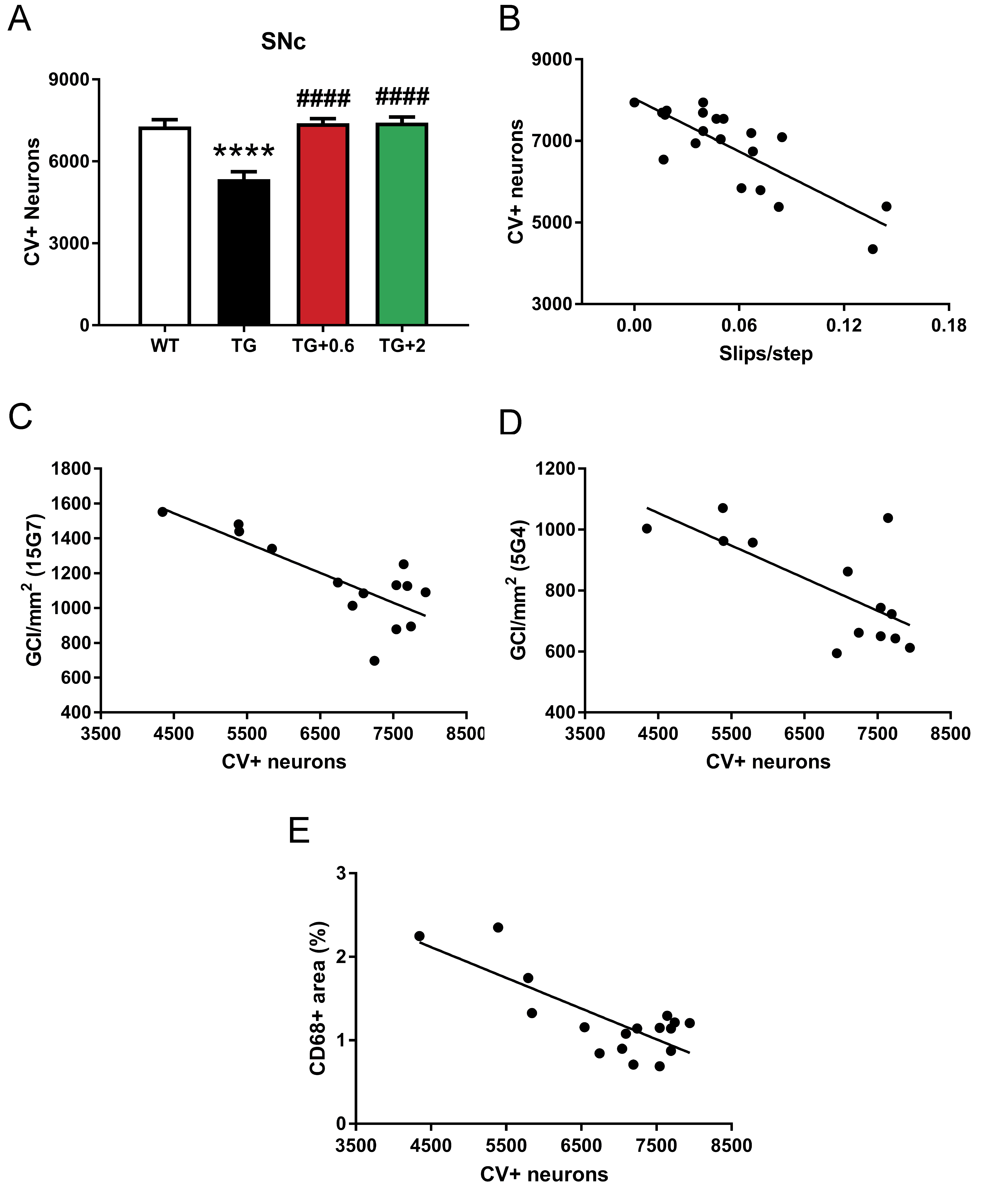

Supplement: Supplementary file 1 — Supporting Information FIG. S1. Anle138b prevents neuronal loss in the SNc of PLP‐hαSyn mice. (A) Stereological counting of the total number of cresyl violet positive (CV+) neurons in SNc . n = 5 per experimental group. Error bars indicate SEM. ANOVA, CV+ neurons/genotype: **** P < 0.0001; CV+ neurons/treatment: #### P < 0.0001 (Bonferroni's test). (B) Correlation analysis of number of neurons in the SNc and number of slips per step. P < 0.0001; R2 = 0.6478. (C,D) Correlation analysis of the number of CV+ neurons in the SNc and the density of GCIs (15G7 and 5G4 respectively) in the SNc. P = 0.0010, R2 = 0.6076 and P = 0.0079, R2 = 0.4884 respectively. (E) Correlation analysis of number of CV+ neurons in the SNc and CD68+ area in the SN. P = 0.0002; R2 = 0.6051. [file MDS-34-255-s001.tif]

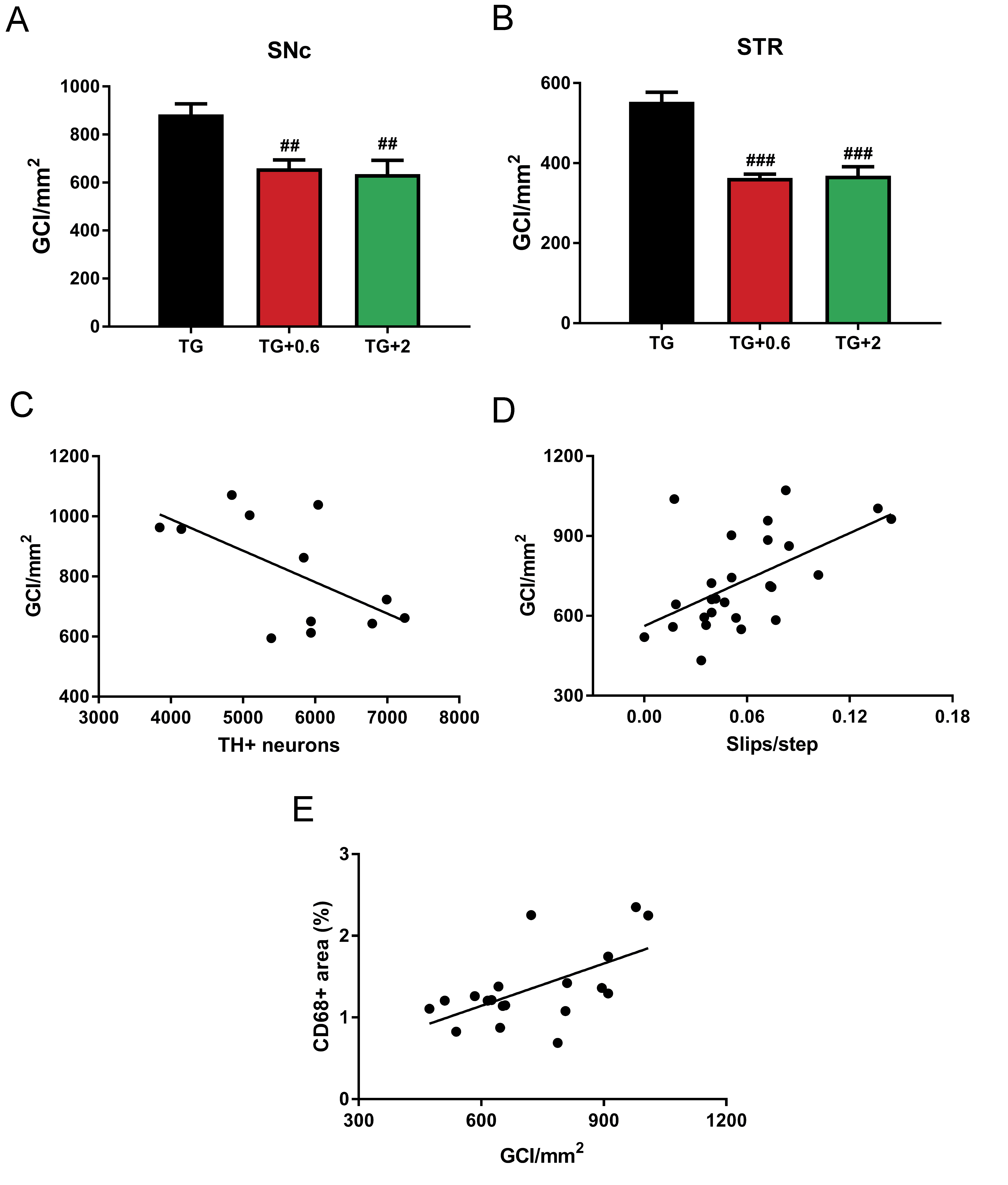

Supplement: Supplementary file 2 — Supporting Information FIG. 2. Anle138b reduces α‐syn accumulation and microglial activation in PLP‐hαSyn mice. (A,B) GCI density in SNc and STR of PLP‐hαSyn mice determined by stereological counting of brain sections stained with the 5G4 antibody against oligomeric human α‐syn and expressed in GCI/mm2. n = 8 to 9 per experimental group. Error bars indicate SEM. ANOVA, GCI density/treatment: ## P < 0.01; ### P < 0.001 (Bonferroni's test). (C) Correlation analysis of density of GCIs (5G4) in the SNc and number of TH+ neurons in the same region. P = 0.0368; R2 = 0.3672. (D) Correlation analysis of density of GCIs (5G4) in the SNc and number of slips per step. P = 0.0026; R2 = 0. 3194. (E) Correlation analysis between the CD68+ area in the SN and the density of GCIs (5G4) in the same brain region. P = 0. 0079; R2 = 0. 3477. [file MDS-34-255-s002.tif]
